# Utilization of Provider-Initiated HIV Testing and Counseling as an Intervention for PMTCT Services Among Pregnant Women Attending Antenatal Clinic in a Teaching Hospital in Ethiopia

**DOI:** 10.3389/fpubh.2019.00205

**Published:** 2019-07-24

**Authors:** Begashaw Melaku Gebresillassie, Yohannes Kelifa Emiru, Daniel Asfaw Erku, Amanual Getnet Mersha, Abebe Basazn Mekuria, Asnakew Achaw Ayele, Henok Getachew Tegegn

**Affiliations:** ^1^School of Pharmacy, College of Medicine and Health Sciences, University of Gondar, Gondar, Ethiopia; ^2^School of Medicine, College of Medicine and Health Sciences, University of Gondar, Gondar, Ethiopia

**Keywords:** pregnant women, utilization, provider-initiated HIV testing and counseling, PMTCT, Ethiopia

## Abstract

**Purpose:** Little is known about acceptance of provider-initiated HIV testing and counseling (PICT) as an intervention for prevention of mother to child transmission of HIV (PMTCT) in many parts of sub-Saharan Africa including Ethiopia. This study aimed at assessing the utilization and acceptance rate of PICT as an intervention for PMTCT among pregnant women attending University of Gondar referral and teaching hospital (UoGRTH), Ethiopia.

**Methods:** A hospital-based cross-sectional study was conducted on 364 pregnant women attending antenatal care clinic at UoGRTH through an interviewer-administered questionnaire. Frequencies, means, and percentages were used to report different variables. Univariate analysis and multivariate logistic regression analysis were used to come up with factors associated with acceptance of PICT services.

**Results:** Out of 364 respondents, 298 330 (81.7%) of them accepted provider-initiated HIV testing and counseling. Rural residency (AOR: 364, 95% CI: 2.17–6.34), higher educational status (AOR: 3.15, 95% CI: 1.86–6.82), planning of HIV test disclosure to male partners (AOR: 7.81, 95% CI: 3.17–13.14), and a higher average monthly income (AOR: 4.01, 95% CI: 2.32–7.61) were found to be strong predictors of acceptance of provider-initiated HIV testing and counseling.

**Conclusions:** The present study revealed a higher rate of acceptance of PICT among pregnant women. Enhancing access to and consistent use of antenatal care service among pregnant women and encouraging the active involvement of male partners are recommended to further increase the uptake of provider-initiated HIV testing and counseling.

## Background

Recently, children and adolescents have been given a higher priority for HIV preventive and treatment services owing to a higher prevalence of undiagnosed HIV infection in these population compared to adults (1). Mother-to-child transmission of HIV (MTCT) represents one of the biggest public health problem and continues to account for a considerable percentage of new cases of HIV infections among Ethiopian children (2). Only in 2012, more than 750,000 people were living with HIV with an estimated 37,600 were children, around 20,000 were newly infected and more than 22,000 were pregnant women (3). Without any interventions, the risk of HIV transmission from mother to child ranges from 5 to 10% during gestation, 10 to 20% during delivery, and 10 to 20% through infant feeding (4, 5). MTCT of HIV can be prevented through early detection of maternal HIV and administration of antiretroviral prophylaxis infection during pregnancy (6).

The national prevention of mother-to-child transmission of HIV (PMTCT) guideline of Ethiopia is based on the four-pronged approaches and promotes the integration of PMTCT and HIV counseling and testing services within the family planning and reproductive health services (7). Provider-initiated HIV counseling and testing (PICT) services are routinely provided freely to all pregnant women attending antenatal and post-natal services. In 2011, the proportion of pregnant women counseled and tested for MTCT in Ethiopia was 33.4%, and only 9.3% of infants born to HIV positive mothers received ARV prophylaxis for PMTCT (8). A number of factors have been identified which contribute to the low level of utilization of PICT services. Less frequent ANC attendance and follow up, a higher rate of home delivery and lost from follow up are some of the major reasons (9–13).

In order to reduce the number of infants with HIV infection, there has been a rapid and intensive scale-up of ARV prophylaxis and provider-initiated HIV counseling and testing (PICT) services in the country since 2005 (14). Yet, regardless of intensive interventions to scale-up, the coverage and utilization of PMTCT services in Ethiopia remains low. The aim of this study was to assess the utilization and acceptance rate of provider-initiated HIV testing and counseling (PICT) as an intervention for PMTCT of HIV among pregnant women attending antenatal clinic of University of Gondar teaching and referral hospital, northwest Ethiopia.

## Materials and Methods

### Study Design and Setting

This was a hospital-based quantitative cross-sectional study conducted on 364 pregnant women attending antenatal care (ANC) clinic at University of Gondar Referral and Teaching Hospital (UoGRTH), Ethiopia. UoGRTH, the only referral center in the area with multiple specialized clinics including ANC follow up clinic, is located in Gondar town, northwest Ethiopia. The hospital provides ANC service for more than 15,000 pregnant women annually. The study was conducted from January 1 to March 30, 2018. This study was approved by the ethical review committee of School of Pharmacy, University of Gondar. Written informed consent was also secured from all the respondents before commencing this study.

### Sampling and Recruitment Strategies

All pregnant women attending ANC clinic of UoGRTH were taken as a source population and those pregnant mothers who visit the ANC clinic during the study period were our study population. We used a single population proportion formula taking into account a 95% confidence interval, 5% margin of error and 80% rate of acceptance and utilization of PICT (15) and 5% was considered for possible non-response rate to come up with a final sample size of 423. As per the information we got from Gondar university referral hospital Statistics and Information Office, an estimated number of 25 pregnant women visit the ANC clinic of UoGRTH on a daily basis. Hence, the proportion of pregnant women who will visit the ANC clinic during the data collection period was estimated to be 7. Accordingly, a systematic random sampling technique was utilized and every fourth pregnant woman available at the ANC clinic and who met the inclusion criteria during the 2-month data collection period were included. All pregnant women were interviewed in the waiting room before meeting with the doctor.

### The Study Tools

The data were collected performed five well-trained nurses working at ANC clinic via interviewer-administered questionnaire. Data collectors were trained intensively on contents of the questionnaire, data collection methods and ethical concerns by the principal investigator. The overall data completeness and consistency was checked by the principal investigator on a daily basis. The questionnaire was created by modifying items in three previously used instruments regarding the acceptance and utilization of PICT services (12, 15, 16) and items were thoroughly reviewed for relevance by a team of experts including obstetrics and gynecology specialists along with a reproductive health expert. The survey instrument was further validated by pre-testing on 25 pregnant women who were not included in the final analysis and relevant modifications were done before the start of final data collection. The final data collection tool included questions assessing demographic and pregnancy-related information including age, highest level of education, history and number of ANC visits, knowledge of PMTCT, and acceptability of PITC services, knowledge, and risk perception on HIV/AIDS, attitude toward PITC and perceived benefit of HIV test, and partner's feedback for HIV positive test result.

### Statistical Analysis

The final data collected were analyzed using Statistical Package for the Social Sciences (SPSS) software version 21.0 for Windows (SPSS Inc., Chicago, IL). Frequencies, means, and percentages were used to report different variables. Univariate analysis and multivariate logistic regression analysis were used to come up with factors associated with acceptance and utilization of PICT services. The results were adjusted for patients' demographic and pregnancy-related characteristics and OR with 95% CI were computed along with corresponding *p*-value (*p* < 0.05) as cut off points for determining statistical significance among different variables.

## Results

### Socio-Demographic Characteristics

From a total of 423 pregnant women invited to participate, 364 of them completed the survey giving a response rate of 89.8%. The mean age of respondents was 26 years with a standard deviation of ± 5.0. Majority of the respondents were Orthodox Christians (87.6%) and urban residents (79.7%). About two-thirds (66.2%) of the subjects drawn in this study were in their third trimester of pregnancy. The socio-demographic and pregnancy-related characteristics of respondents are summarized in Table 1.

**Table 1:** Socio-demographic characteristics and factors associated with acceptance of PICT among respondents, 2018 (*N* = 364).

**Variables**	**PICT acceptance, N (%)**			
	**Yes (*N* = 298)**	**No (*N* = 66)**	***p*-value**	**AOR (95% CI)**
**Age group, in years**			0.121	
<20	50 (16.8)	12 (18.2)		–
21–30	172 (57.7)	39 (29.1)		–
>30	76 (25.5)	15 (22.7)		–
**Residence**			0.001^**^	
Rural	205 (68.8)	37 (56.1)		3.64 (2.17–6.34)
Urban	93 (31.2)	29 (43.9)		1(ref)
**Educational status**			0.003^**^	
Illiterate	71 (23.8)	22 (33.3)		3.15 (1.86–6.82)
Primary	70 (23.5)	14 (21.2)		2.73 (1.17–5.43)
Secondary	95 (31.9)	19 (28.8)		1.89 (0.40–2.05)
Tertiary	62 (20.8)	11 (16.7)		1(ref)
**Religion**			0.413	
Orthodox Christian	190 (63.7)	36 (54.5)		–
Muslim	89 (29.9)	21 (31.8)		–
Others^*^	19 (6.4)	9 (13.6)		–
**Employment status**			0.020^**^	
Government employed	66 (22.1)	15 (22.8)		0.72 (0.31–1.42)
Self-employed	60 (20.1)	22 (33.3)		0.31 (0.12–0.85)
Unemployed	172 (57.7)	29 (43.9)		1(ref)
**Average monthly family income**			0.001^**^	
<100	193 (64.8)	39 (59.1)		1(ref)
100–150	69 (23.1)	18 (27.3)		2.91 (1.31–5.66)
>150	36 (12.1)	9 (13.6)		4.01 (2.32–7.61)
**Parity**			0.712	
1–2 children	191 (64.1)	28 (42.4)		–
3–4 children	77 (25.8)	27 (40.9)		–
>4 children	30 (10.1)	11 (16.7)		–
**Partner reaction to a positive result**			0.010^**^	
Positive	202 (67.8)	39 (59.1)		1.07 (0.89–2.01)
Negative	96 (32.2)	27 (40.9)		1(ref)
**Planned to disclose results to partner after testing**			0.001^**^	
Yes	263 (88.2)	54 (81.8)		7.81 (3.17–13.14)
No	35 (11.8)	12 (18.2)		1(ref)
**Attitude toward PICT**			0.041^**^	
Positive (favorable)	241 (80.9)	41 (62.1)		–
Negative (ow favorable)	57 (19.1)	25 (37.9)		–

* *Protestant, Jehovah witness*.

** *Significant association (p-value <0.05). AOR, Adjusted odds ratio; CI, Confidence interval*.

Protecting their children from getting the infection (64.4%) and concern for their own health (44.3%) were most frequent reasons given for accepting PICT, while anticipating the negative reaction from their partners for HIV test result (43.9%) and not being ready for conducting HIV test (33.3%) as the common reasons for not accepting PICT (Table 2).

**Table 2:** Reasons for acceptance and refusal of PICT among respondents, 2018 (*N* = 364).

**Variables**	**Frequency (%)**
**REASONS FOR ACCEPTANCE (*****N*** **=** 298)^*^	
To protect my child from getting HIV	192 (64.4)
To protect my partner from getting HIV	46 (15.4)
To know my HIV status	132 (44.3)
**REASONS FOR REFUSAL (*****N*** **=** 66)^*^	
Fear of stigma and discrimination	13 (19.7)
Fear of partner's reaction for HIV positive result	29 (43.9)
Fear of knowing HIV positive test result	12 (18.2)
Not ready for conducting HIV test	22 (33.3)
Lack of HIV risk perception	9 (13.6)
Need for partner's consent	19 (28.8)

* *More than option is possible*.

### Prevalence and Factors Associated With Acceptance of PICT

Out of 364 respondents, 298 330 (81.7%) of them accepted provider-initiated HIV testing and counseling. Those variables that were significantly associated with acceptance of PICT in the bivariate analysis were further examined in multivariate logistic regression. Accordingly, residency, educational status, planning of HIV test disclosure to male partners, and average monthly income found to have a significant association in multivariate logistic regression analysis. Accordingly, the odds of acceptance of PICT was 3.64 times higher among rural residents as compared to urban residents (AOR: 364, 95% CI: 2.17–6.34). Pregnant women who had no formal education were 3.15 times more likely to accept PICT than those who attended tertiary education (AOR: 3.15, 95% CI: 1.86–6.82). Similarly, pregnant women who had average monthly income higher than 150 USD were 4.01 times more likely to accept PICT than those who had average monthly income of <100 USD (AOR: 4.01, 95% CI: 2.32–7.61). Respondents who planned to disclose their HIV status to their partners were 7.81 times (AOR: 7.81, 95% CI: 3.17–13.14) more likely to accept PICT than those who did not plan to disclose their results. No significant associations were found between PICT acceptance with age group, religion, marital status, and parity both in the bi-variant and multivariate logistic regression analysis.

## Discussion

In our study, the acceptance of PICT was 81.9%, which is comparable with the studies done in different regions of Ethiopia including Assossa (80.8%) (15), and Arba-Minch (74.4%) (17). However, a much lower acceptance rate of PICT was reported in Illubabor (27.0%) (18). The variation in acceptability of PICT is also pronounced in different parts of African countries with acceptability ranging from 79% in Zimbabwe, 88.3% in Cameroon, 95% in Botswana, 97% in Uganda and 99.9% in Zimbabwe (16, 19–22). The relatively higher acceptance rate in our study could be partially explained by the fact that most pregnant women consider that PICT as a standard care for PMTCT and due to the high availability and affordability of comprehensive HIV/AIDS care in the past couple of years through community sensitization and enhancing the accessibility of on-site rapid HIV testing.

Both bi-variant and multivariate regression analysis were done to identify factors associated with acceptance of PICT among women attending ANC follow up. Accordingly, Women who had no formal education were more likely to accept PICT than those who attended tertiary education. Similarly, the odds of acceptance of PICT was 3.64 times higher among rural residents as compared to urban residents. This could be partially explained by the fact that women who are illiterates or unschooled might accept PICT due to fear of the negative response from their physicians or health care providers and their relatively low possibility of asking their physicians regarding medical advice (23, 24). Furthermore, enhancing PICT acceptance may be achieved at the cost of pregnant women not knowing that PICT is optional, which might be also the case in our study (25). A similar study conducted in Botswana reported that about 68% of women felt that they could not refuse PICT (26).

Pregnant women who had a higher average monthly income were also more likely to accept PICT than those who had low average monthly income, which might be attributed to the fact that women with lower average monthly income are less able to make autonomous decisions as they are financially dependent and are not empowered economically. Furthermore, acceptance of PITC is increased as the number of ANC visits increases in our study, which corroborates with the finding of the study done in Zimbabwe (16). One possible explanation for this is the fact that frequent visit of health centers and close contact with health care providers increase the possibility of acquiring knowledge about PICT, PMTCT, and other preventive strategies. Moreover, women who did not plan to disclose their test results to their partner refused to test. This could be due to a number of reasons including fear of separation, cultural influence and anticipated lack of acceptance as reported in other similar studies (15).

## Limitations

The study has some limitations that should be taken into account while interpreting the results. As the study is cross-sectional and depends on self-reported assessment, underreporting is very likely. A larger-scale and multi-centered survey that includes more diverse participants is needed to provide more accurate findings. Regardless of the above limitations, the study provides useful information that will inform the implementation and consolidation of PICT to PMTCT Amhara and other regions of Ethiopia.

## Conclusions

The present study revealed a higher level of utilization of PICT among pregnant women attending antenatal care clinic at University of Gondar referral and teaching hospital, Ethiopia. A higher number of antenatal care visits, urban residency, and a higher level of knowledge on prevention of mother to child transmission of HIV are associated with a higher acceptance rate of provider-initiated HIV testing and counseling. Enhancing access to and consistent use of antenatal care service among pregnant women is recommended to further increase the uptake of provider-initiated HIV testing and counseling. Physicians working in ANC clinics should promote and encourage the active involvement of partners during ANC services to reduce the trouble that pregnant women face to disclose their HIV test result. Further studies using mixed methods (both qualitative and quantitative approach) is recommended at a national level to come up with other factors associated with the acceptance and utilization of PICT.

## Ethics Statement

This study was approved by the ethical review committee of School of pharmacy, University of Gondar with a reference number of SoP-UoG-134/2018. Written informed consent was also secured from all the respondents before commencing this study.

## Author Contributions

All authors listed have made a substantial, direct and intellectual contribution to the work, and approved it for publication.

### Conflict of Interest Statement

The authors declare that the research was conducted in the absence of any commercial or financial relationships that could be construed as a potential conflict of interest.
